# Micro fluorescence *in situ* hybridization (μFISH) for spatially multiplexed analysis of a cell monolayer

**DOI:** 10.1007/s10544-016-0064-0

**Published:** 2016-04-30

**Authors:** D. Huber, J. Autebert, G. V. Kaigala

**Affiliations:** IBM Research – Zurich, Säumerstrasse 4, 8803 Rüschlikon, Switzerland

**Keywords:** Fluorescence *in situ* hybridization, Microfluidic probe, Spatial multiplexing, Microfluidics

## Abstract

We here present a micrometer-scale implementation of fluorescence *in situ* hybridization that we term μFISH. This μFISH implementation makes use of a non-contact scanning probe technology, namely, a microfluidic probe (MFP) that hydrodynamically shapes nanoliter volumes of liquid on a surface with micrometer resolution. By confining FISH probes at the tip of this microfabricated scanning probe, we locally exposed approximately 1000 selected MCF-7 cells of a monolayer to perform incubation of probes — the rate-limiting step in conventional FISH. This method is compatible with the standard workflow of conventional FISH, allows re-budgeting of the sample for various tests, and results in a ~ 15-fold reduction in probe consumption. The continuous flow of probes and shaping liquid on these selected cells resulted in a 120-fold reduction of the hybridization time compared with the standard protocol (3 min vs. 6 h) and efficient rinsing, thereby shortening the total FISH assay time for centromeric probes. We further demonstrated spatially multiplexed μFISH, enabling the use of spectrally equivalent probes for detailed and real-time analysis of a cell monolayer, which paves the way towards rapid and automated multiplexed FISH on standard cytological supports.

**Electronic supplementary material:**

The online version of this article (doi:10.1007/s10544-016-0064-0) contains supplementary material, which is available to authorized users.

## Introduction

**Fig. 1:**
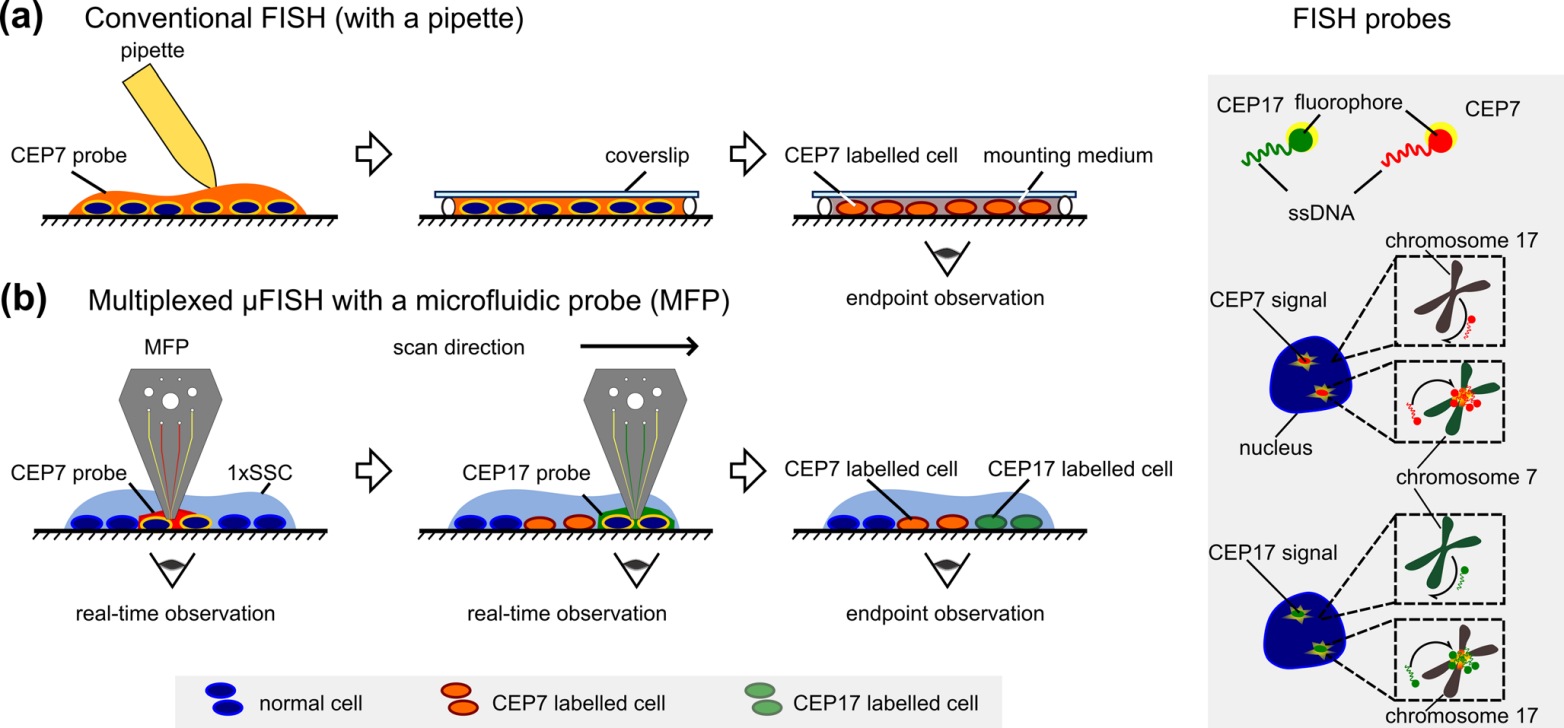
Schematic of key process steps in conventional FISH and in μFISH***.*** **a** Using a pipette in conventional FISH, CEP7 probes are deposited on the entire cell monolayer. After post-processing, an endpoint observation is made. **b** In μFISH implemented with an MFP, CEP7 probes are localized on selected cells, and the FISH signal is inspected in real time. After incubation, the liquid is switched in the MFP head, scanning is performed, and different cells are exposed to CEP17 probes. FISH probes specific to the centromeric region of chromosome 7 and chromosome 17 were used in **a** and **b**

*In situ* hybridization (ISH) is an important class of cytogenetic techniques, allowing high-resolution detection, quantification, and localization of nucleic acid (NA) targets*.* ISH is performed without isolation of the targets from their source, i.e., *in situ*, and is widely used in research and diagnostics. It relies on the sequence-specific hybridization of probes to their complementary targets in individual cells, followed by direct or indirect detection of the labelled probe. ISH was first demonstrated by Gall and Pardue (1969) using radioactive rRNA probes to visualize extrachromosomal rDNA. Later, the first non-radioisotopic ISH was demonstrated by Manning et al. (1975) using rRNA-biotin probes and an avidin-based detection system*.* A key milestone in ISH-based techniques was fluorescence *in situ* hybridization (FISH) (Bauman et al. 1980). This technique allowed the direct and simultaneous detection of multiple targets. Advances in fluorescence microscopy, fluorescent dyes, biotechnology, bioinformatics, and research on the human genome project in the late 90’s accelerated the development of methods to synthesize and design NA probes for FISH. Currently, a range of probes can be synthesized to locate and quantify specific short RNAs, genes, entire chromosomes, and even cells (Evanko 2007). Thus, the convergence of several factors has made FISH a standard cytogenetic technique for nuclear studies in diagnostics and research (on average, 2.73 FISH papers were published per day in the past 20 years^1^). In this paper, we present a new method to implement micrometer-scale interphase FISH that we term μFISH. μFISH enables a rapid nucleic acid analysis, and we demonstrate its usefulness for spatially multiplexed FISH, Fig. 1.

Multiplexed FISH is often used to locate multiple targets in a cell nucleus simultaneously using probes of different colors, in order to study sparse cytological substrates (Lichter 1997). However, the number of spectrally distinct fluorophores for multiplexed FISH is limited. To visualize additional targets simultaneously, fluorescent probes of different fluorescence intensities were used to distinguish the complete set of human chromosomes *in situ* (Tanke et al. 1999). Quantum dots (QD) allow the resolution of even more colors as they have narrow emission bands with minimal spectral overlap (Pathak et al. 2001). However, QD are bulky (15–20 nm), and QD-DNA constructs have a lower mobility than free DNA (Ioannou et al. 2009) and are therefore seldom used for direct detection of targets in FISH (Zhang et al. 2013).

Despite the merits of FISH-based approaches, their widespread use for diagnostics is limited for several reasons. Conventional implementations of FISH comprise multiple steps, such as sample-specific enzymatic treatment, fixation, denaturation, manual pipetting of probes, incubation, post-hybridization washes to remove unbound probes, and endpoint visualization, Fig. 1. Those steps are mostly performed manually, are time consuming, and require experienced personnel. In addition, FISH remains an expensive test owing to the high price of probes. Further, expensive imaging platforms are necessary to visualize spectrally distinct probes for multiplexed FISH. To make FISH assays more pervasive in diagnostics, there is therefore a need to reduce the cost per test, introduce automation, and simplify the implementation of multiplexed analysis.

A few microfluidic platforms were developed for instance, to automate FISH analysis (Sieben et al. 2008; Tai et al. 2013) and to increase throughput (Sieben et al. 2007). Within such platforms, interphase FISH has been integrated with a range of analytical techniques, such as flow cytometry (Liu et al. 2011), chemistrode (Liu et al. 2009), Förster resonance energy transfer (FRET) (Packard et al. 2011) and immunostaining (Zhang et al. 2006). Microfluidics-based devices have also been used to analyze specific chromosomal translocations within spread metaphases on slides (Vedarethinam et al. 2010; Shah et al. 2011). In the context of diagnostics, devices primarily comprising closed microfluidic channels or reservoirs have been developed to miniaturize FISH assays for prenatal diagnostics (Ho et al. 2012) and the analysis of circulating tumor cells (Lim et al. 2012; Mottet et al. 2014; Perez-Toralla et al. 2015; Gogoi et al. 2016) or cancer malignancies (Zanardi et al. 2010; Kurz et al. 2011; Mughal et al. 2014). All these implementations need cell immobilization within microfluidic systems or surface treatment to ensure cell adhesion. Other microfluidic implementations are suitable for cell suspensions (Zhang et al. 2006; Liu et al. 2009; Liu et al. 2011; Packard et al. 2011), but unsuitable for the analysis of cytological samples forming monolayers, such as adherent cells, fixed cytological samples and tissue sections. Working with such adherent biological substrates is vital as the retrieval of immobilized cells from their substrate prior to flowing them in closed channels may introduce nuclear damage, thereby impeding hybridization and subsequently the result itself. Towards using microfluidics for tissue section analysis, Histo Flex (Søe et al. 2011) implemented localized and multiplexed RNA FISH by placing an elastomeric lid patterned with microfluidic channels on tissue sections and then flowing reagents to perform local FISH on certain cells. However, this approach limits the flexibility in adapting to morphological variations between samples.

In this paper, we demonstrate an interactive and versatile implementation of μFISH on cell monolayers at the micrometer-scale using a microfluidic probe (MFP). The MFP is a non-contact, scanning probe technology that localizes nanoliter volumes of liquids on substrates using hydrodynamic flow confinement (HFC) (Kaigala et al. 2011). With this technology, we can precisely localize (bio)chemicals on standard biological substrates in the “open space”, i.e., without the need for inserting the biological entities into closed channels. Prior demonstrations with MFPs (Queval et al. 2010; Ainla et al. 2012; Sarkar et al. 2014) are micro-immunohistochemistry on tissue sections (Lovchik et al. 2012), cell inactivation (Kaigala et al. 2011), local lysis of cells for expression analysis and cell patterning (Kashyap et al. 2016, Rapid subtractive patterning of live cell layers with a microfluidic probe, unpublished), and high-quality protein patterning (Autebert et al. 2016). In this work, we specifically use a vertically-oriented probe head and hierarchical HFC (hHFC) to expose a cell monolayer to multiple processing liquids simultaneously (Autebert et al. 2014). The localization and scanning capabilities of this technology allow incubation of FISH probes on selected cells on a monolayer. We also perform spatially multiplexed μFISH, which cannot be performed using conventional FISH protocols as the latter entail covering the entire cell monolayer with the probe mix, Fig. 1. In this spatially multiplexed μFISH, we incubate spectrally equivalent probes on distinct areas of a cell monolayer, and visualize the FISH signals with a simplified detection system.

## Materials and methods

### MFP head and platform

**Fig. 2:**
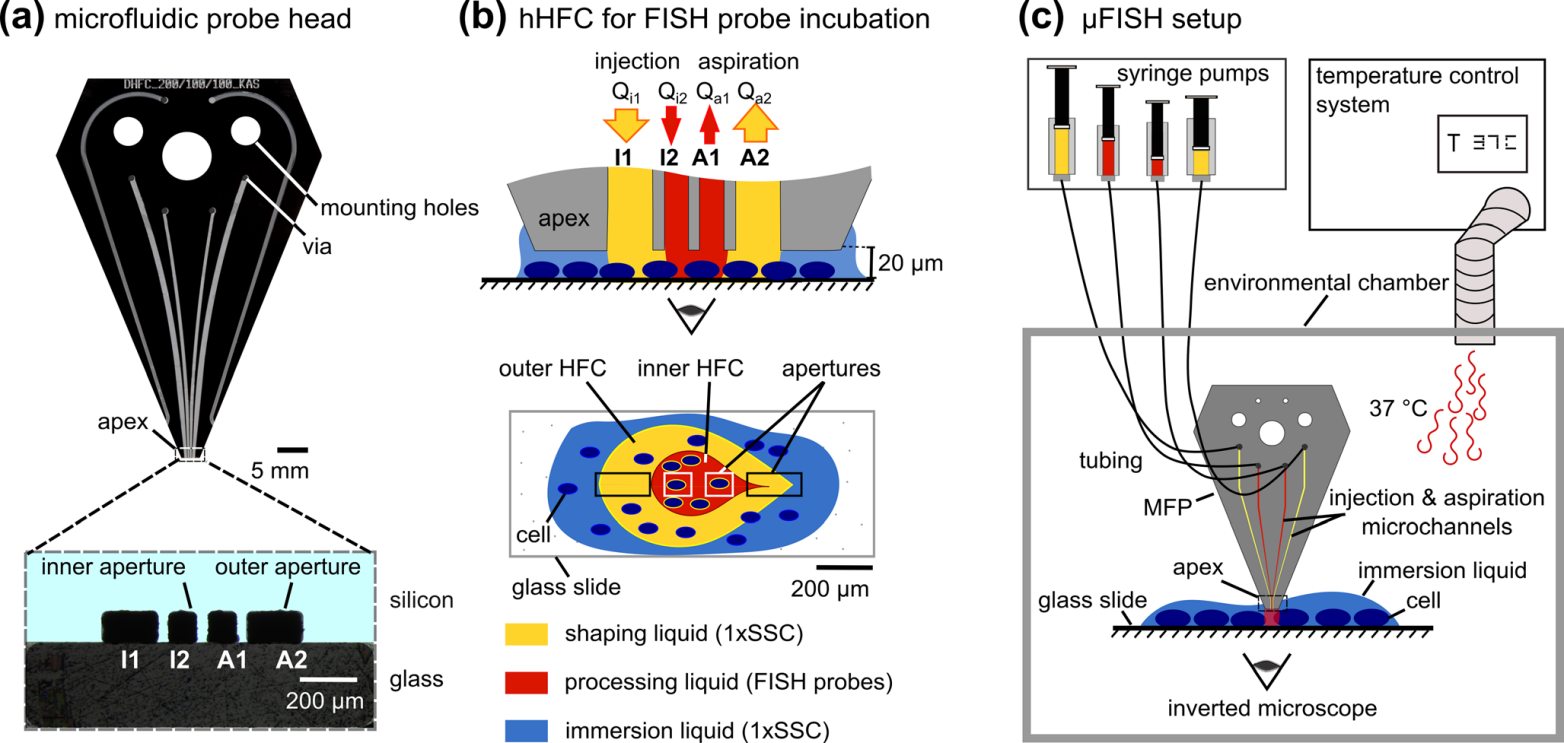
μFISH implemented with the microfluidic probe. The MFP head **a** comprising four apertures is connected to the fluidic systems to create an hierarchal HFC **b** with FISH probes in the inner HFC. **c** The entire MFP platform is placed in an environmental chamber

The microfabricated MFP head is a key component of the platform and contains the channels for fluid flow, Fig. 2a. The microfabrication of the silicon–glass hybrid heads has been described elsewhere (Kaigala et al. 2011). The design comprises six channels, two for injection, two for aspiration and two outer channels to replenish the immersion liquid on the substrate without direct interaction. The inner channels, etched to a depth of 100 μm, converge towards the apex and form four apertures I1, I2, A1 and A2, with the outer aperture dimensions 100 × 200 μm^2^ (I1 and A2, with flow rates *Q*_i1_, *Q*_a2_) and the inner aperture dimensions 100 × 100 μm^2^ (I2 and A1, with flow rates *Q*_i2_, *Q*_a1_). To establish an hHFC, the flow rules are conventionally set to be *Q*_i2_ = |*Q*_a1_| and |*Q*_a1_ + *Q*_a2_| = 3 × (*Q*_i1_ + *Q*_i2_) (Autebert et al. 2014). Here, these flow rules were set to account for the viscous FISH probes in the processing liquid, and the flow rates were accordingly set to 1, 0.2 μL min^−1^ (*Q*_i1_, *Q*_i2_) and −0.2, −2 μL min^−1^ (*Q*_a1_, *Q*_a2_), Fig. 2b.

The MFP platform comprises linear stages to control the position of the head relative to the substrate, syringe pumps, an environmental chamber, and a microscope, Fig. 2c. The head was mounted to the *z*-stage of the platform via a mounting plate and linked to the syringes (Hamilton, 1705 TLLX) with a connector (Dolomite, 8-ports circular connector), tubing and adapters (IDEX, Tygon). The stages are computer-controlled and motorized (Lang GmbH, Hüttenberg, Germany), with a sub-micrometer accuracy. The apex-to-surface distance as well as the *x-* and *y-*coordinates were monitored using the encoded position of the stage. Flow rates were set with Nemesys pumps (Cetoni GmbH, Korbussen, Germany) and Qmix software (Cetoni GmbH, Korbussen, Germany). 1000 μL and 250 μL syringes were filled with 1× saline sodium citrate (SSC) (from 20× SSC stock, 3.0 M NaCl, 0.3 M sodium citrate, pH 7.0, Thermo Fisher Scientific, Cat. No. 15557**-**044). An environmental chamber (Life Imaging Services GmbH, “The Cube and the Box”) enclosed the platform and the microscope, Supplemental Fig. S1.

### Cell handling and preparation

MCF-7 cells (American Type Culture Collection (ATCC), HTB-22, breast adenocarcinoma) were cultured as recommended by ATCC. Cells were seeded in chamber slides (Thermo Fisher Scientific, Cat. No. 10717931) at a density of 10^5^**–**10^6^ cells cm^−2^. After 2**–**3 days, the monolayer was washed with phosphate-buffered saline (PBS, pH 7.4, Thermo Fisher Scientific, Cat. No. 10010-023) and heat immobilized at 82 °C for 2 min. Next, the cells were rinsed with PBS and digested using pepsin at 37 °C for 8–10 min (Leica Biosystems, LK-101 A). Subsequently, they were rinsed with 2× SSC and fixed using Carnoy’s fixative (ethanol: acetic acid 3:1 (v/v). Sigma Aldrich, Cat. No. 02860 and 537010) at 4 °C for 40 min. Finally, the cells were dried for 5 min at room temperature (RT), washed with 2× SSC for 1 min twice, and then renatured in 2× SSC at 37 °C for 20 min.

### Conventional FISH protocol

The compartments of the chamber slides were removed, and 10 μL FISH hybridization mix was deposited onto the cells (2 μL FISH probes in 8 μL FISH hybridization buffer). Buffer (KBI-FHB) and centromeric probes with a Platinum *Bright550* dye (KBI-20017R and KBI-20007R) were purchased from Leica Biosystems. These cells with the probes were coverslipped (Menzel, Braunschweig, Germany). Probes and chromosomes were denatured at 75 °C for 5**–**10 min and then incubated at 37 °C in a dark, humidified chamber. The coverslip was removed, and non-specifically bound probes on the chamber slide were removed by washing twice with 0.1 % IGEPAL CA**-**630 in 2× SSC (v/v) for 1 min at RT and with 0.3 % IGEPAL CA-630 in 0.4× SSC (v/v) at 72 °C for 2 min (Sigma Aldrich, Cat. No. I8896). Subsequently, an additional wash was performed with 1× SSC at RT for 1 min. The cells were then mounted with mounting medium containing DAPI (Thermo Fisher Scientific, Cat. No. S36938) for inspection.

### μFISH protocol

The compartments of the chamber slides were removed, the cells were immersed in 10 μL FISH buffer, sealed with a coverslip, and the chromosomes were denatured at 75 °C for 5–10 min in FISH hybridization buffer. 5 μL FISH hybridization mix (Conventional FISH protocol section) was denatured separately at 75 °C for 5 min in a PCR tube (VWR, Cat. No. 20170-012). Hoechst dye was added to the probes at a concentration of 0.2 μg mL^−1^ (Thermo Fisher Scientific, Cat. No. H3570). These probes were pipetted onto a sheet of parafilm and aspirated into the inner aperture (I2) of the head. Concurrently, the coverslip was removed from the chamber slide, and the cells were immersed in 1× SSC. This chamber slide was transferred to the sample holder of the MFP platform, and the head was positioned ~20 μm above the monolayer. The flow confinement was established using the flow rules defined in “MFP head and platform” section. The probes described above were injected from I2, and 1× SSC was injected from I1. Aspiration of probes and 1× SSC was performed from A1 and A2, respectively, Fig. 2b. After 10 min interaction of the hHFC with the cells (equivalent to 10 min incubation), *Q*_i2_ and *Q*_a1_ were stopped, and the cells were washed with 1× SSC flowing between the outer apertures for 2 min (*Q*_i1_ and *Q*_a2_). The head was positioned away from the slide before imaging. For multiplexing, the head was positioned away from substrate after the first probe hybridization wash, and 20 μL of 1× SSC was purged from A1 and I2, and 50 μL from A2 and I1, to avoid cross-contamination of the different probes used. Subsequently, the probe-loading procedure explained above was repeated.

### Image acquisition and processing

Both the endpoint observation for conventional FISH and the real-time observation for μFISH were performed using an inverted microscope at 10×, 40× and 60× magnification (Nikon Eclipse Ti-E with objectives CFI Plan Fluor DLL 10×, ELWD 40× and ELWD 60×, respectively). An LED lamp (Sola, Lumencor) was used for illumination. Image acquisition was performed using an ORCA-flash 4.0 camera. For imaging, the NIS Elements Basic Research software (Nikon Instruments Europe, V.4.0) was used. Brightness and contrast adjustments of raw images as well as merging were done using the open-source FIJI (ImageJ) software (http://fiji.sc/Fiji), Supplemental Fig. S4.

## Results and discussion

**Fig. 3:**
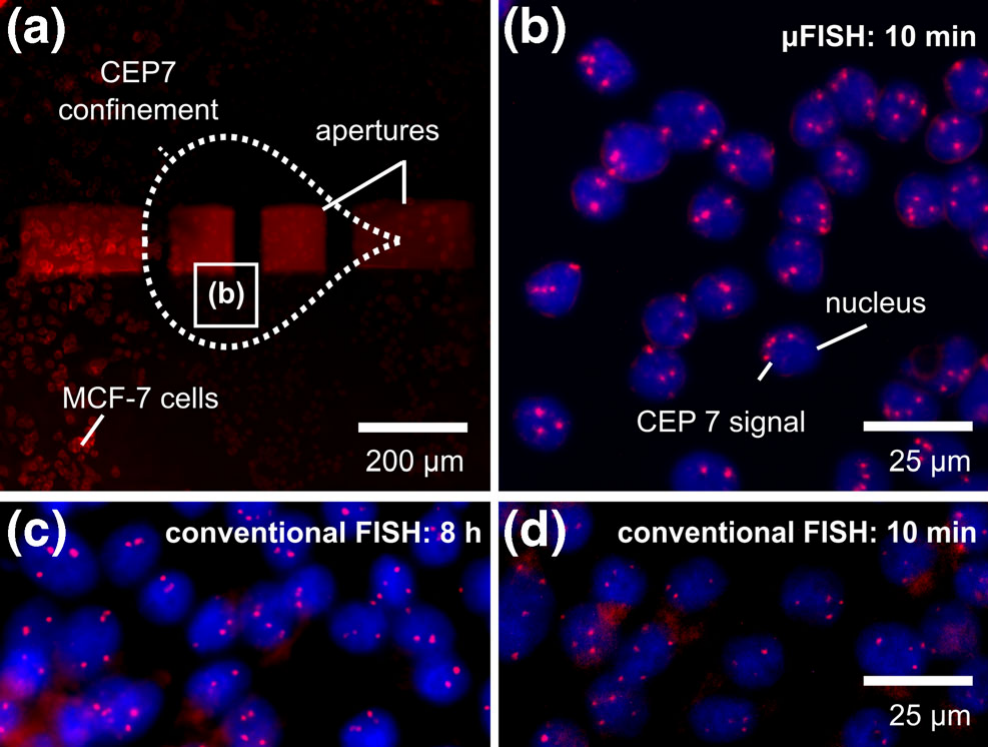
Conventional FISH and μFISH with the MFP on an MCF-7 cell monolayer. **a** Fluorescence micrograph showing the four apertures, with the dashed line outlining the confined processing liquid in contact with MCF-7 cells. The processing liquid contains CEP7 probes. **b** Fluorescence micrograph after μFISH was performed on MCF-7 cells with a 10 min incubation, showing multiple FISH signals specific to centromere 7 (*red*) in each nuclei (*blue*). **c**, **d** Fluorescence images of FISH signals after incubation of 8 h and 10 min with conventional FISH

We performed the sequence-specific hybridization of probes, a key step in FISH, using the MFP. As a the model system, we used an immobilized MCF-7 cell monolayer and the centromeric FISH probes (satellite enumeration probes) CEP7 and CEP17 to visualize chromosome 7 and 17, Fig. 1. We chose centromere-specific probes because they are used in diagnostics, for example, in the assessment of the HER2 status in breast-cancer cells. In particular, CEP17 probes are used to normalize the *ERBB2* gene to the chromosome 17 counts. A subpopulation of cells in the monolayer was incubated with probes, Fig. 3. The MFP head used in this work has two inner apertures of 100 × 100 μm^2^ that are spaced by 50 μm. Using this head, we localized the probes to a footprint (area) of ~0.096 mm^2^, equivalent to the area of about 1000 cells. We detected a FISH signal in this subpopulation of cells and found no detectable signal beyond this footprint. In contrast, in conventional FISH, the entire monolayer is covered with probes during the incubation step, Fig. 1a and b.

A specific challenge to implement μFISH with the MFP is the viscosity of the probe mix. The FISH probe mix is viscous, largely owing to the presence of dextran sulfate, which serves to increase the effective probe concentration and consequently improves the hybridization efficiency (Lederman et al. 1981). Localizing the viscous probe mix with the MFP has two important implications. First, the localization of a liquid at the apex of the head is due to the non-symmetric flow rates between the injection and aspiration apertures (*Q*_a, tot_  ≥ 3 × *Q*_i, tot_) resulting in a continuous flow of the viscous processing liquid on the cell monolayer. This interaction of the localized viscous processing liquid exerts increased shear (shear increases linearly as a function of viscosity), which leads to delamination of the cell monolayer. To prevent this delamination of cells, we operated the MFP at 0.2 μL min^−1^, the lowest flow rate our fluidic system permitted. Second, at these low flow rates, we increased the injection and aspiration flow rates of the shaping liquid (*Q*_i1_, *Q*_a2_) to ensure that the viscous processing liquid remains confined. By operating the MFP under these conditions, we were able to confine the viscous processing liquid and to prevent cell delamination, even when incubation was performed for an extended duration of 2 h. We noted that diluting the probe mix with 1× SSC reduces the viscosity, but results in an increased incubation time.

A useful attribute of the MFP-based μFISH implementation is the ability to observe the FISH signal in real time. We observed centromere-specific FISH signals within 3 min of initiating the probe incubation step (Supplemental Fig. S3) with the MFP, which is approximately a 120-fold reduction in the incubation time recommended by the probe supplier (>6h). This 3 min incubation translates to a probe consumption of 0.6 μL with *Q*_i2_ of 0.2 μL min^−1^. Although surface coverage in μFISH is different from conventional FISH, up to 16 slides could be processed using our device, with 3 min incubation each and a total volume of 10 μL FISH probes, which is the volume used in conventional FISH for a single slide. Moreover, we compared the FISH signals under three different conditions (Fig. 3b): (a) conventional FISH with 8 h incubation, (b) conventional FISH with 10 min incubation, and (c) μFISH with 10 min incubation. We observed that the μFISH results with 10 min MFP-based incubation were better (higher intensity of spots and lower background) than those for 10 min of conventional incubation (Fig. 3d) and comparable to those of 8 h of conventional incubation (Fig. 3c). We hypothesize that the reduced incubation time is largely due to the continuous replenishment of the FISH probe mix on the cell monolayer. In contrast, in conventional FISH the probes diffuse overnight on top of the cells, resulting in a depletion layer, further lowering the concentration of probes accessing the cells.

Independently, using our MFP-based FISH implementation, we also observed a significant reduction in the cell-rinsing time compared with conventional rinsing. We substituted a single rinsing step for conventional rinsing methods comprising three 6-min steps and including detergents. At the end of probe incubation, we stopped the flow of FISH probes in the inner two apertures, whereas the flow of shaping liquid (SSC) continued. This continuous flow of SSC removes unbound probes within 1 min of rinsing, resulting in a very low background. Conventional rinsing uses detergents that can alter the structural integrity of cell membranes. Efficient rinsing with the MFP obviates the use of such detergents, thereby leaving the cytoskeleton minimally affected, which might be critical in certain applications, and eliminates the need for further manual manipulations of the cells prior to observation.

**Fig. 4:**
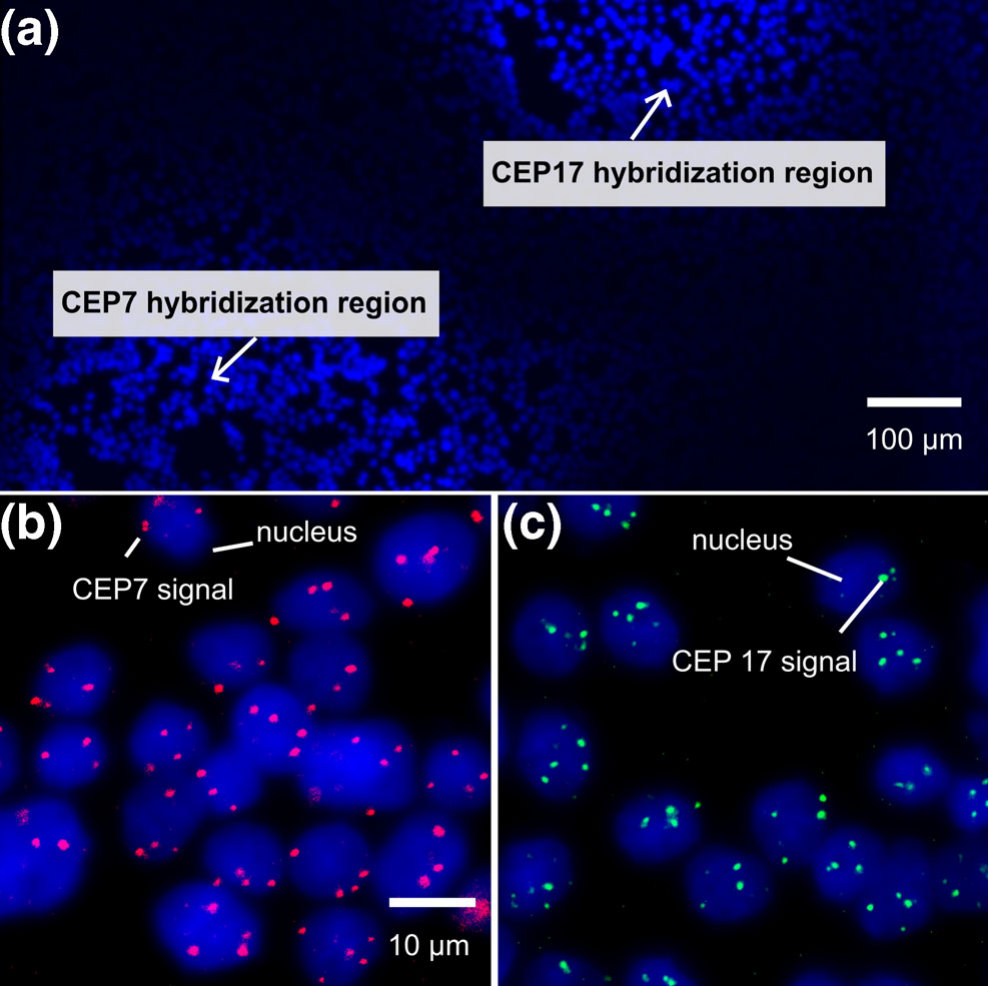
Fluorescence images of spatially multiplexed μFISH with the MFP. **a** Two regions of the MCF-7 monolayer were chosen for spatially multiplexed μFISH: **b** Hybridization region with CEP7 signal (*red*) and **c** hybridization region with CEP17 signal (*green*) in nuclei (*blue*)

Spatially multiplexed μFISH is particularly relevant to resolve multiple targets simultaneously. We chose two probes (CEP7 and CEP17), and incubated them on two distinct areas on the MCF-7 cell monolayer (Fig. 4). The footprints chosen were spaced by ~200 μm (Fig. 4a), and within them cells were incubated for 10 min with CEP7 and CEP17 probes, respectively. The dyes on the CEP7 and CEP17 probes were spectrally equivalent. We obtained comparable results for μFISH signals with 10 min MFP-based incubation (Fig. 3b) and for multiplexed μFISH results with 10 min MFP-based incubation (Fig. 4b and c). The implication of this spatially multiplexed μFISH is that, in practice, it may now be possible to multiplex a large number of FISH probes on a single substrate, e.g., by sampling and performing different tests across different regions of a heterogeneous tissue sample. Moreover, because of the precise localization of the FISH signal, it allows the use of spectrally equivalent probes, whereas in conventional FISH, probe dyes have to be spectrally distinct. Thus, a low-cost, mono-wavelength imaging device can now potentially be used to observe multiplexed μFISH.

## Concluding remarks

The μFISH implementation demonstrated here is a versatile technique to perform rapid, spatially multiplexed analysis of nucleic acids in adherent cells. This technique is characterized by significantly shorter incubation time, has better probe budgeting and utilization, is compatible with other steps in the standard workflow of conventional FISH (Supplemental Fig. S2), and most importantly, facilitates performing simultaneous tests on a given budget of sample. Probe consumption can be further reduced by implementing liquid recirculation, i.e., by circulating the probes back and forth between the inner apertures of the head as described in (Autebert et al. 2016). However, we note that recirculation of viscous probes within the MFP is yet to be explored and may impose special considerations on the fluid-handling system.

Specifically, MFP-based μFISH with CEP7 probes on MCF-7 cells resulted in ~120-fold reduction of hybridization time compared with a conventional FISH implementation, likely due to convective-enhanced transport of probes and more efficient rinsing than in conventional FISH implementations. Approaches to improve these characteristics include optimizing flow rates, pinching the flow confinement, including agents to influence molecular crowding, and optimizing the temperature, for example. Further, the ability to perform real-time observation makes it feasible to periodically measure the evolution of the FISH signal and estimate optimal signal-to-background values.

In our experiments, we used an MFP head design that results in an HFC footprint of ~0.096 mm^2^, which translates into a coverage of ~1000 cells on the surface. We chose to interact with 1000 cells as this is statistically representative of a multiplexed cytological smear. This HFC footprint area can be scaled up to the millimeter-scale with some modifications of the size and design of apertures (unpublished work). More interesting, performing μFISH with the MFP on fewer cells in order to get a higher resolution spatial profile across as sample is likely to be useful in the context of selected research questions. In terms of microfabrication of the heads, apertures sizes of 2 × 2 μm^2^ have been demonstrated (Kaigala et al. 2011) that could result in footprints of 10 μm^2^; however, a challenge observed in preliminary experiments is that channels of small dimensions are prone to clogging with cell debris during operation. This can be overcome by liquid shaping-based solutions (Autebert et al. 2014) combined with a reasonable reduction in aperture size. Nonetheless, with the current HFC footprint dimensions, it is theoretically possible to perform several hundred individual tests on a cytological sample of an area of 1 cm^2^ using spectrally equivalent probes, which in practice could still be done on a timescale of hours owing to the short incubation times and better rinsing with the MFP-based μFISH. In our demonstration, we successfully exposed distinct footprints sequentially to distinct FISH probes, aided by manual switching of the probes. In addition, multiple regions on the monolayer could be exposed to different processing liquids in parallel using a head with multiple pairs of apertures for multiple flow confinements. We believe that such a multiplexed analysis will significantly improve the range of cytological tests feasible not only on cell monolayers, but also on tissue sections, resulting in improved and more accurate tissue profiles for diagnosis. Finally, μFISH, which can be used with any type of FISH probe, allows assessing a wide variety of targets within one sample. We are confident that in addition to DNA-level *in situ* analysis of biological samples, it can, for example, be used in the future for combined quantification of RNA, and therefore provide a comprehensive profile for detailed cytological analysis.

## Electronic supplementary material

ESM 1 (DOCX 2729 kb)

## Footnotes

1 Web of Science, query “fluorescence *in situ* hybridization” (from 18.02.2016).

## References

[CR1] Ainla A, Jeffries GDM, Brune R, Orwar O, Jesorka A (2012). A multifunctional pipette. Lab Chip.

[CR2] Autebert J, Kashyap A, Lovchik RD, Delamarche E, Kaigala GV (2014). Hierarchical hydrodynamic flow confinement: efficient use and retrieval of chemicals for microscale chemistry on surfaces. Langmuir.

[CR3] Autebert J, Cors J, Taylor D, Kaigala GV (2016). Convection-enhanced biopatterning with hydrodynamically confined nanoliter volumes of reagents. Anal. Chem..

[CR4] Bauman JG, Wiegant J, Borst P, van Duijn P (1980). A new method for fluorescence microscopical localization of specific DNA sequences by *in situ* hybridization of fluorochromelabelled RNA. Exp. Cell Res..

[CR5] D. Evanko (2007) Fully cooked FISH. In: Nat. Milestones. http://www.nature.com/milestones/miledna/pdf/miledna03.pdf

[CR6] Gall JG, Pardue ML (1969). Formation and detection of RNA-DNA hybrid molecules in cytological preparations. Proc. Natl. Acad. Sci..

[CR7] Gogoi P, Sepehri S, Zhou Y, Gorin MA, Paolillo C, Capoluongo E, Gleason K, Payne A, Boniface B, Cristofanilli M, Morgan TM, Fortina P, Pienta KJ, Handique K, Wang Y (2016). Development of an automated and sensitive microfluidic device for capturing and characterizing circulating tumor cells (CTCs) from clinical blood samples. PLoS One.

[CR8] Ho SSY, Chua C, Gole L, Biswas A, Koay E, Choolani M (2012). Same-day prenatal diagnosis of common chromosomal aneuploidies using microfluidics-fluorescence *in situ* hybridization. Prenat. Diagn..

[CR9] Ioannou D, Tempest HG, Skinner BM, Thornhill AR, Ellis M, Griffin DK (2009). Quantum dots as new-generation fluorochromes for FISH: an appraisal. Chromosom. Res..

[CR10] Kaigala GV, Lovchik RD, Drechsler U, Delamarche E (2011). A vertical microfluidic probe. Langmuir.

[CR11] Kurz CM, Moosdijk SVD, Thielecke H, Velten T (2011). Towards a cellular multi-parameter analysis platform: fluorescence *in situ* hybridization (FISH) on microhole-array chips. Conf. Proc. IEEE Eng. Med. Biol. Soc..

[CR12] Lederman L, Kawasaki ES, Szabo P (1981). The rate of nucleic acid annealing to cytological preparations is increased in the presence of dextran sulfate. Anal. Biochem..

[CR13] Lichter P (1997). Multicolor FISHing: what’s the catch?. Trends Genet..

[CR14] Lim LS, Hu M, Huang MC, Cheong WC, Gan ATL, Looi XL, Leong SM, Koay ES-C, Li M-H (2012). Microsieve lab-chip device for rapid enumeration and fluorescence *in situ* hybridization of circulating tumor cells. Lab Chip.

[CR15] Liu W, Kim HJ, Lucchetta EM, Du W, Ismagilov RF (2009). Isolation, incubation, and parallel functional testing and identification by FISH of rare microbial single-copy cells from multi-species mixtures using the combination of chemistrode and stochastic confinement. Lab Chip.

[CR16] Liu P, Meagher RJ, Light YK, Yilmaz S, Chakraborty R, Arkin AP, Hazen TC, Singh AK (2011). Microfluidic fluorescence *in situ* hybridization and flow cytometry (μFlowFISH). Lab Chip.

[CR17] Lovchik RD, Kaigala GV, Georgiadis M, Delamarche E (2012). Micro-immunohistochemistry using a microfluidic probe. Lab Chip.

[CR18] Manning JE, Hershey ND, Broker TR, Pellegrini M, Mitchell HK, Davidson N (1975). A new method of *in situ* hybridization. Chromosoma.

[CR19] Mottet G, Perez-Toralla K, Tulukcuoglu E, Bidard F-C, Pierga J-Y, Draskovic I, Londono-Vallejo A, Descroix S, Malaquin L, Louis Viovy J (2014). A three dimensional thermoplastic microfluidic chip for robust cell capture and high resolution imaging. Biomicrofluidics.

[CR20] Mughal F, Baldock SJ, Karimiani EG, Telford N, Goddard NJ, Day PJR (2014). Microfluidic Channel-assisted screening of hematopoietic malignancies. Genes Chromosom. Cancer.

[CR21] M. M. Packard, Shusteff M., Alocilja E. (2011) Novel, rapid DNA-based on-chip bacterial identification system combining dielectrophoresis and amplification-free fluorescent resonance energy transfer assisted in-situ hybridization (FRET-ISH). In: Mohseni H, Agahi MH, Razeghi M (eds) Proceedings of SPIE, Bellingham. p 80990 J

[CR22] Pathak S, Choi SK, Arnheim N, Thompson ME (2001). Hydroxylated quantum dots as luminescent probes for *in situ* hybridization. J. Am. Chem. Soc..

[CR23] Perez-Toralla K, Mottet G, Guneri ET, Champ J, Bidard F-C, Pierga J-Y, Klijanienko J, Draskovic I, Malaquin L, Viovy J-L, Descroix S (2015). FISH in chips: turning microfluidic fluorescence *in situ* hybridization into a quantitative and clinically reliable molecular diagnosis tool. Lab Chip.

[CR24] Queval A, Ghattamaneni NR, Perrault CM, Gill R, Mirzaei M, McKinney RA, Juncker D (2010). Chamber and microfluidic probe for microperfusion of organotypic brain slices. Lab Chip.

[CR25] Sarkar A, Kolitz S, Lauffenburger DA, Han J (2014). Microfluidic probe for single-cell analysis in adherent tissue culture. Nat. Commun..

[CR26] Shah P, Vedarethinam I, Kwasny D, Andresen L, Skov S, Silahtaroglu A, Tümer Z, Dimaki M, Svendsen WE (2011). FISHprep: a novel integrated device for metaphase FISH sample preparation. Micromachines.

[CR27] Sieben VJ, Debes Marun CS, Pilarski PM, Kaigala GV, Pilarski LM, Backhouse CJ (2007). FISH and chips: chromosomal analysis on microfluidic platforms. IET Nanobiotechnol..

[CR28] Sieben VJ, Debes-Marun CS, Pilarski LM, Backhouse CJ (2008). An integrated microfluidic chip for chromosome enumeration using fluorescence *in situ* hybridization. Lab Chip.

[CR29] Søe MJ, Okkels F, Sabourin D, Alberti M, Holmstrøm K, Dufva M (2011). HistoFlex—a microfluidic device providing uniform flow conditions enabling highly sensitive, reproducible and quantitative *in situ* hybridizations. Lab Chip.

[CR30] Tai C-H, Ho C-L, Chen Y-L, Chen WL, Lee G-B (2013). A novel integrated microfluidic platform to perform fluorescence *in situ* hybridization for chromosomal analysis. Microfluid. Nanofluid..

[CR31] Tanke HJ, Wiegant J, van Gijlswijk RP, Bezrookove V, Pattenier H, Heetebrij RJ, Talman EG, Raap AK, Vrolijk J (1999). New strategy for multi-colour fluorescence *in situ* hybridisation: COBRA: COmbined binary RAtio labelling. Eur. J. Hum. Genet..

[CR32] Vedarethinam I, Shah P, Dimaki M, Tumer Z, Tommerup N, Svendsen WE (2010). Metaphase FISH on a chip: miniaturized microfluidic device for fluorescence *in situ* hybridization. Sensors (Basel).

[CR33] Zanardi A, Bandiera D, Bertolini F, Corsini CA, Gregato G, Milani P, Barborini E, Carbone R (2010). Miniaturized FISH for screening of onco-hematological malignancies. Biotechniques.

[CR34] Zhang Q, Zhu L, Feng H, Ang S, Chau FS, Liu W-T (2006). Microbial detection in microfluidic devices through dual staining of quantum dots-labeled immunoassay and RNA hybridization. Anal. Chim. Acta.

[CR35] Zhang W, Hubbard A, Brunhoeber P, Wang Y, Tang L (2013). Automated multiplexing quantum dots *in situ* hybridization assay for simultaneous detection of ERG and PTEN gene status in prostate cancer. J. Mol. Diagn..

