# Supplementary material for: Micro fluorescence in situ hybridization (μFISH) for spatially multiplexed analysis of a cell monolayer
Source: Biomed Microdevices. 2016 Apr 30;18:40. doi: 10.1007/s10544-016-0064-0 (PMC4853442; doi:10.1007/s10544-016-0064-0)
Supplement: Supplementary file 1 — (DOCX 2729 kb) [file 10544_2016_64_MOESM1_ESM.docx]

Micro fluorescence *in situ* hybridization (µFISH) for spatially multiplexed analysis of a cell monolayer

Deborah Huber, Julien Autebert, Govind Kaigala*

IBM Research-Zurich, Säumerstrasse 4, CH-8803 Rüschlikon, Switzerland


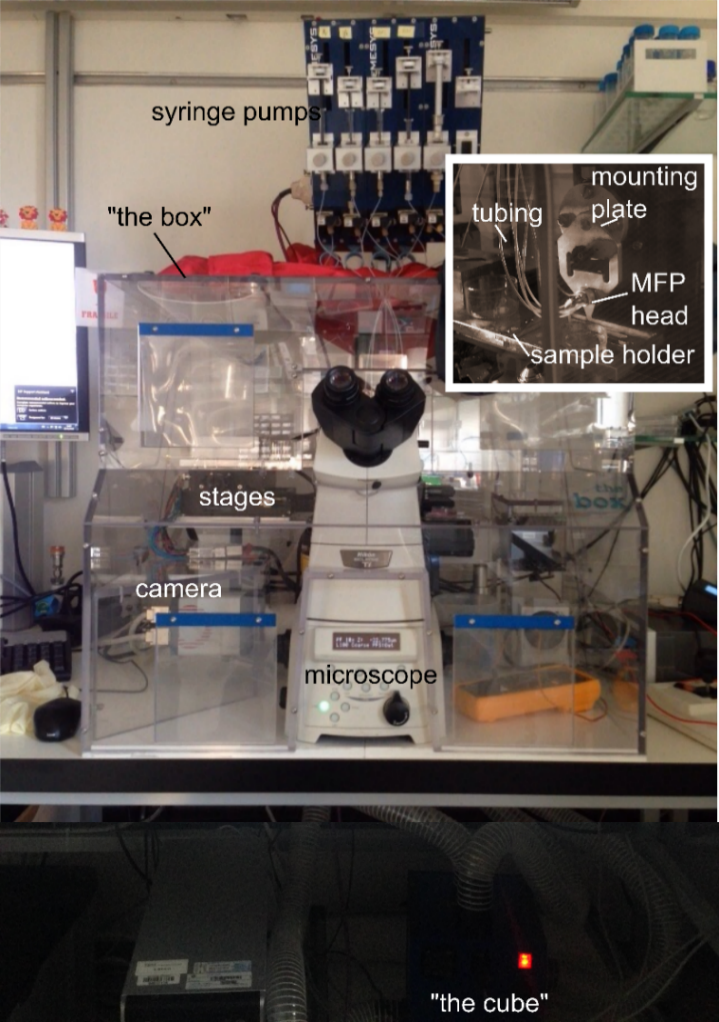
**Supplementary Information**

## Supplementary figure S1: Microfluidic probe platform.

**Fig. S1**

MFP platform. (a)MFP platform comprises high precision motorized XYZ scanning stage, peripherals for handling liquids - pumps, syringes and tubings, and a microfabricated head. This platform is placed on top of an inverted microscope for real-time observation of the local MFP-based FISH hybridization. The environmental chamber (“the box”) surrounds the MFP platform and microscope. Temperature is controlled by “The cube”. (b) The MFP head is installed to the z-stage via the mounting plate and controls the apex-to-substrate gap. The sample on the sample holder is moved along x- and y-axis relative to the MFP head

##
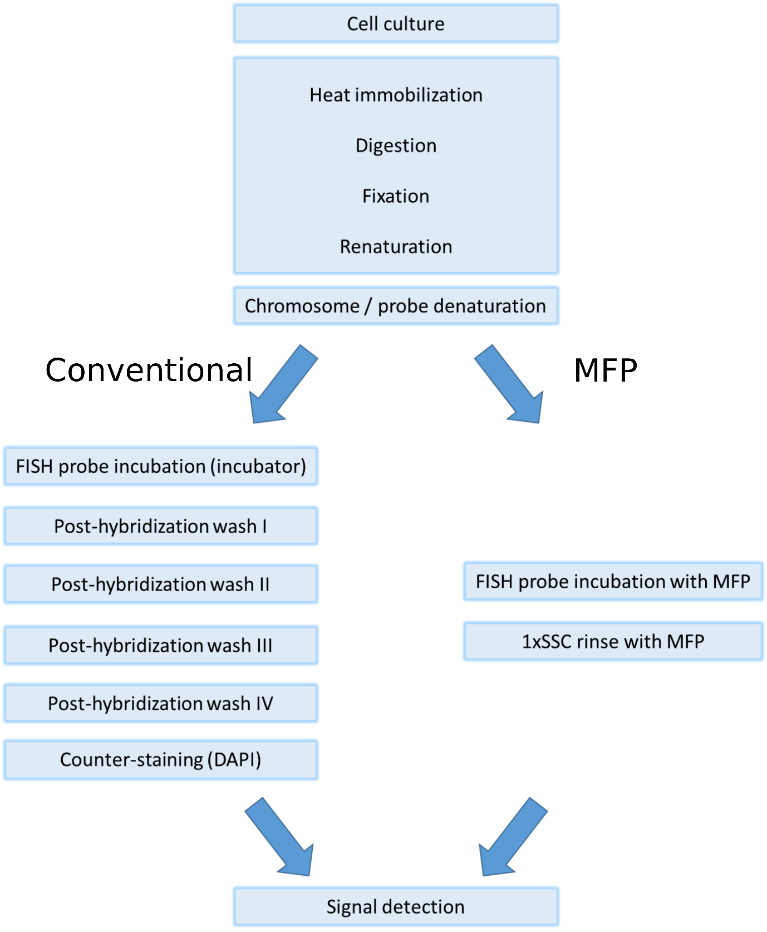
Supplementary figure S2: Detailed workflow of conventional and MFP-based FISH.

**Fig. S2**

In conventional FISH, incubation and washes are performed on-bench and the entire slide/Petri dish. In μFISH, all processes after preparation of the cells are performed on the MFP platform and therefore on the inverted microscope

**Supplementary figure S3:** Real-time image acquisition during incubation in μFISH.


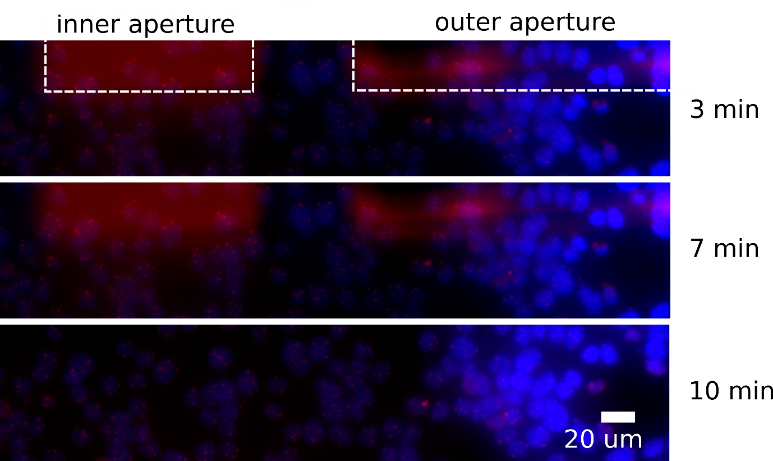


**Fig. S3**

Fluorescence images were taken with a camera at 40× magnification. CEP7 probes (containing Hoechst dye. See section 2.2) were confined on MCF-7 cells, localizing the hybridization reaction to a region on cells of ~0.096 mm^2^. Fluorescence images were taken after 3 min, 7 min, 10 min incubation. FISH signal (red) was excited and detected with TRITC filter sets, the nuclear dye (blue) with the DAPI filter sets

**Supplementary figure S4:** Raw and processed images.


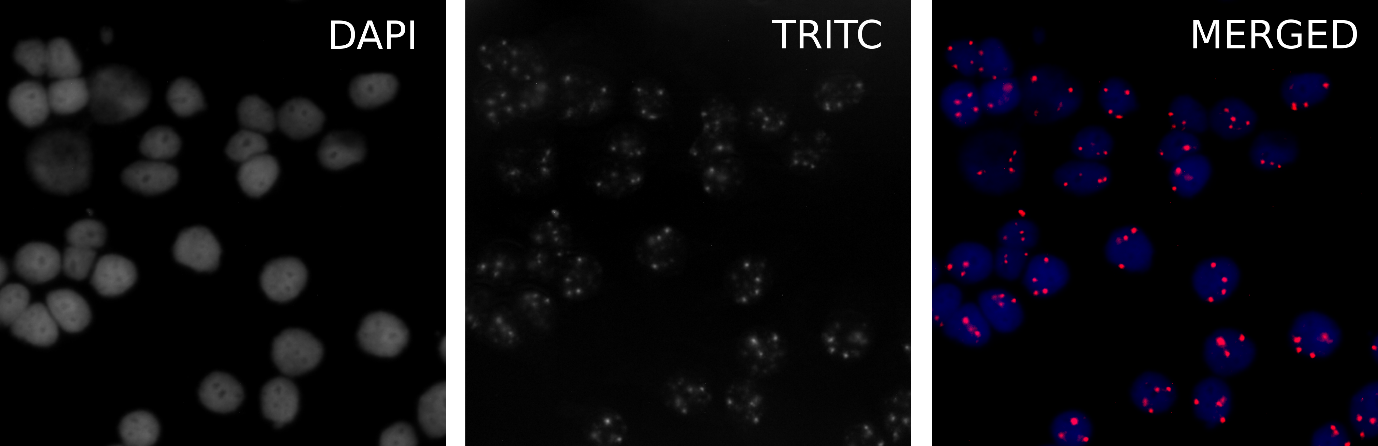


**Fig. S4**

Fluorescence images were taken with a camera at 40× magnification. FISH signal was excited and detected with an LED source and TRITC filter sets, the nuclear dye with the DAPI filter sets and the channels were imaged separately in gray-scale. These raw images were exported to the Fiji software, brightness and contrast were adjusted and then the two images were merged and pseudo-colors were assigned to the images from the TRITC (red) and DAPI (blue) channel
